# Serum sLOX-1 and 25-Hydroxyvitamin D_3_ Across the Glycemic Spectrum: Associations with Metabolic and Microvascular Parameters in Prediabetes and Type 2 Diabetes

**DOI:** 10.3390/jcm15207989

**Published:** 2026-10-15

**Authors:** Maide Hacer Alagoz, Mine Adas, Yasemin Gokden, Gulcan Guntas

**Affiliations:** 1 Department of Clinical Biochemistry, Prof. Dr. Cemil Tascıoglu City Hospital, 34384 Istanbul, Turkey; 2 Department of Internal Medicine, Prof. Dr. Cemil Tascıoglu City Hospital, Health Sciences University, 34384 Istanbul, Turkey; 3 Department of Medical Biochemistry, Atlas University School of Medicine, 34408 Istanbul, Turkey

**Keywords:** diabetes mellitus, prediabetes, sLOX-1, 25-hydroxy vitamin D, microvascular complications

## Abstract

**Background/Objectives:** Oxidative endothelial injury and vitamin D deficiency have independently been implicated in diabetic vascular complications, yet their distribution across the glycemic spectrum, from normoglycemia through prediabetes to established type 2 diabetes, has not been systematically characterised. This study aimed to evaluate serum soluble lectin-like oxidized low-density lipoprotein receptor-1 (sLOX-1) and 25-hydroxyvitamin D_3_ [25(OH)D_3_] levels across the glycemic spectrum and to examine their associations with microvascular complications and metabolic parameters in prediabetic individuals and patients with type 2 diabetes mellitus (T2DM). **Methods:** In this prospectively recruited cross-sectional study, 80 adults were enrolled: 26 healthy controls, 27 prediabetic individuals, and 27 T2DM patients. Serum sLOX-1 was quantified by enzyme-linked immunosorbent assay (ELISA) and 25(OH)D_3_ by liquid chromatography–tandem mass spectrometry (LC-MS/MS). Between-group comparisons were performed using the Kruskal–Wallis test with Dunn’s post hoc correction. Spearman’s rank correlation and binary logistic regression were applied to examine associations with metabolic and microvascular parameters. **Results:** Serum sLOX-1 was significantly elevated in both prediabetic (median 10.75 ng/mL; *p* = 0.0397) and T2DM (8.83 ng/mL; *p* = 0.0209) groups compared to controls (3.50 ng/mL) in unadjusted analyses. After adjustment for age and body mass index (BMI) by quantile regression, the elevation persisted in the prediabetic group (adjusted ratio of medians = 2.91, *p* = 0.040) but was substantially attenuated and no longer significant in the T2DM group (ratio = 1.81, *p* = 0.265). Serum 25(OH)D_3_ was significantly lower in the T2DM group than in prediabetic individuals (12.11 vs. 22.69 ng/mL; *p* = 0.0024) but did not decline progressively across the glycemic spectrum; vitamin D deficiency (<20 ng/mL) was present in 78% of T2DM patients. In binary logistic regression (five predictors, events per variable (EPV) = 5.4), 25(OH)D_3_ (odds ratio (OR) = 0.922, *p* = 0.020) and log-transformed urinary albumin-to-creatinine ratio (logUACR; OR = 4.014, *p* = 0.001) were independently associated with T2DM status after adjustment for age and waist circumference, while sLOX-1 was not significant (OR = 1.058, *p* = 0.136). In unadjusted analyses, sLOX-1 correlated positively with waist circumference and glycated hemoglobin (HbA1c); both associations were no longer statistically significant after age adjustment. **Conclusions:** These findings show that sLOX-1 is elevated at the prediabetic stage independently of age and BMI, whereas the elevation in established T2DM was not robust to covariate adjustment. Lower 25(OH)D_3_ concentrations were associated with T2DM status in logistic regression, but this association was not significant when the reference group was restricted to normoglycaemic controls, indicating that the finding reflects the contrast between T2DM and prediabetic groups rather than a generalised pattern across the glycaemic spectrum. The two biomarkers were not significantly correlated with one another. Given the cross-sectional design, low EPV, and exploratory nature of all analyses, prospective validation in larger age-matched cohorts is required.

## 1. Introduction

Diabetes mellitus (DM) is a chronic metabolic disorder characterised by persistent hyperglycemia, which results from defects in insulin secretion or insulin resistance, or both [1]. Chronic hyperglycemia causes structural and functional alterations in multiple organs, particularly affecting the vascular endothelium, which plays a central role in the development of diabetic complications [2].

Type 2 diabetes mellitus (T2DM) represents the most common form of diabetes and is associated with a high burden of morbidity and mortality, largely due to its vascular complications [2,3]. These complications are broadly classified as macrovascular and microvascular. Microvascular complications, including diabetic nephropathy, retinopathy, and neuropathy, are primarily driven by chronic hyperglycemia-induced endothelial dysfunction and oxidative stress [3]. Notably, increasing evidence suggests that vascular damage begins long before the clinical onset of overt diabetes, during the prediabetic stage, where metabolic disturbances such as insulin resistance, low-grade inflammation, and oxidative stress are already present [4,5,6].

Prediabetes is an intermediate metabolic state between normoglycemia and diabetes. It is not only associated with an increased risk of progressing to T2DM, but also with early endothelial dysfunction and subclinical vascular damage [4].

Endothelial dysfunction is widely recognized as an early and critical event in the pathogenesis of diabetic vascular disease. Hyperglycemia-induced oxidative stress plays a key role in this process by promoting the formation of reactive oxygen species (ROS), which reduce nitric oxide (NO) bioavailability [7]. Furthermore, chronic hyperglycemia activates several biochemical pathways, including advanced glycation end-product (AGE) formation, protein kinase C (PKC) activation, and the polyol pathway, all of which contribute to endothelial injury and microvascular damage [7,8].

One of the key mediators of oxidative endothelial damage is oxidised low-density lipoprotein (oxLDL), which exerts its biological effects primarily by binding to the scavenger receptor lectin-like oxidized low-density lipoprotein receptor-1 (LOX-1), expressed on endothelial cells, macrophages, and smooth muscle cells [9,10]. Activation of LOX-1 triggers a cascade of pro-inflammatory and pro-atherogenic signalling pathways, including the activation of nuclear factor-kappa B (NF-κB), increased ROS production, the expression of adhesion molecules and endothelial apoptosis [11]. These mechanisms play a central role in vascular inflammation, remodelling, and the progression of microvascular and macrovascular complications in diabetes.

The soluble form of LOX-1 (sLOX-1) is generated through the proteolytic cleavage of the membrane-bound receptor and is then released into circulation, where it has been associated with endothelial activation [11]. Elevated levels of circulating sLOX-1 have been reported in various pathological conditions, including acute coronary syndromes, metabolic syndrome and diabetes mellitus [10,11,12]. Consequently, sLOX-1 has been proposed as a potential biomarker for the early detection of vascular injury and risk stratification in metabolic diseases.

In recent years, vitamin D has gained increasing attention due to its pleiotropic effects beyond bone metabolism. The vitamin D receptor (VDR) and the activating enzyme CYP27B1 are expressed in β-cells, immune cells, and vascular smooth muscle cells, which suggests that vitamin D plays an important role in glucose metabolism, immune regulation, inflammation and vascular function [13,14]. There is recent evidence that vitamin D deficiency is highly prevalent in patients with prediabetes and T2DM and is associated with increased insulin resistance and poor glycemic control [15,16]. In addition, vitamin D deficiency has been associated with the development and progression of diabetic microvascular complications, such as nephropathy and retinopathy, potentially through anti-inflammatory, anti-fibrotic, and endothelium-protective mechanisms [17,18]. Moreover, vitamin D deficiency has been reported to be closely associated with enhanced vascular oxidative stress in both normal and diabetic models [19].

Despite these independent lines of evidence, few studies have simultaneously examined sLOX-1 and vitamin D levels in the context of early stages of glycemic impairment, particularly in prediabetic individuals. While previous studies have established the role of sLOX-1 as a cardiovascular biomarker [20], detailed its proteolytic regulation [21], and demonstrated its elevation under diabetic conditions [22], the most closely related work to date evaluated circulating oxLDL, sLOX-1, and PCSK9 levels in relation to carotid intima-media thickness in prediabetic and T2DM patients but focused exclusively on macrovascular endpoints [23], leaving the association between sLOX-1, vitamin D status, and microvascular complications largely unexplored across the dysglycemic spectrum. Because nephropathy and retinopathy typically become clinically apparent in established diabetes, we assessed these microvascular endpoints in the T2DM group. In particular, whether elevated circulating sLOX-1 levels are already present at the prediabetic stage, and how they relate to vitamin D status, has yet to be systematically evaluated. The aim of this study was therefore to evaluate serum sLOX-1 levels in individuals with prediabetes and T2DM, to assess its association with metabolic parameters and microvascular complications, and to investigate its relationship with 25-hydroxyvitamin D_3_ [25(OH)D_3_] levels.

## 2. Materials and Methods

### 2.1. Study Design and Participants

This was a cross-sectional, prospective study in which participants were enrolled sequentially over a defined recruitment period, with clinical and biochemical data collected at a single time point. The study was conducted at the Endocrinology and Metabolism Diseases outpatient clinic of Istanbul Prof. Dr. Cemil Tascioglu City Hospital. A total of 80 adults aged 29–69 years were enrolled: 26 healthy controls (Group 1), 27 prediabetic individuals (Group 2), and 27 T2DM patients (Group 3). Prediabetes and T2DM were diagnosed according to 2017 American Diabetes Association (ADA) criteria [24]. Individuals without a history of diabetes and with glycated hemoglobin (HbA1c) 4.6–5.6% constituted the control group, and were recruited from hospital staff at the same institution. Prediabetic and T2DM participants were recruited consecutively from patients attending the outpatient clinic. Exclusion criteria included type 1 DM, secondary causes of hyperglycemia, end-stage renal disease, active malignancy, chronic inflammatory or autoimmune disease, pregnancy, and use of vitamin D or corticosteroid supplements within the preceding 3 months. Following ethics committee approval, recruitment and sample collection were carried out between November 2017 and January 2018, ensuring that seasonal effects on 25(OH)D_3_ concentrations were comparable across groups. All participants provided written informed consent and the study was approved by the local ethics committee (Date: 24 October 2017; Issue: 730) in accordance with the Declaration of Helsinki.

### 2.2. Clinical and Anthropometric Measurements

Body weight, height, waist circumference, and blood pressure were measured using standardised protocols. Blood pressure was recorded but not included in the present analysis. Body mass index (BMI) was calculated as weight/height^2^ (kg/m^2^). Diabetic complications were evaluated in Group 3: nephropathy was defined as a UACR ≥ 30 mg/g creatinine from a single spot urine specimen, and retinopathy was assessed by dilated fundoscopic examination performed by a qualified ophthalmologist and classified dichotomously as present or absent; a standardised severity grading system was not applied. Repeated measurements were not obtained and this classification should therefore be regarded as provisional.

### 2.3. Laboratory Methods

Venous blood was collected after an 8 h overnight fast. Fasting plasma glucose, albumin, creatinine, total cholesterol (TC), triglycerides (TG), HDL-cholesterol, and LDL-cholesterol were determined by a photometric method on a Beckman Coulter AU5821 automated clinical chemistry analyser using Beckman Coulter reagent kits (Beckman Coulter Inc., Brea, CA, USA). HbA1c was measured by ion-exchange high-performance liquid chromatography (HPLC) (Bio-Rad Variant II, Bio-Rad Laboratories, Marnes-la-Coquette, France). Serum sLOX-1 was quantified using a commercial sandwich enzyme-linked immunosorbent assay (ELISA) kit (Human LOX-1 ELISA Kit, Catalogue No. E-EL-H1635; Elabscience Biotechnology Inc., Houston, TX, USA), with a detection range of 0.16–10 ng/mL and a sensitivity of 0.10 ng/mL. Prior to analysis, all serum samples were diluted 1:5 with the sample diluent provided by the manufacturer and the final concentrations were calculated by multiplying by the dilution factor, in accordance with the manufacturer’s instructions. The samples were allowed to clot for 2 h at room temperature, centrifuged at 1000× *g* for 15 min at 2–8 °C, and the supernatants were stored at −80 °C for up to 3 months until analysis, in accordance with the manufacturer’s stated sample stability. Optical density was read at 450 nm, and concentrations were derived from a standard curve. Standards were assayed in duplicate, and the samples were analysed within a single assay batch. The intra-assay coefficient of variation was <10%. 25(OH)D_3_ was measured by liquid chromatography-tandem mass spectrometry (LC-MS/MS) (Zivac ONH-100A, Zivac Technology, Kocaeli, Turkey), the reference method for vitamin D quantification. Total 25(OH)D_3_ was quantified; 25(OH)D_2_ was not separately measured. Vitamin D status was classified per the Turkish Endocrinology and Metabolism Society: severe deficiency <10 ng/mL, deficiency <20 ng/mL, insufficiency 20–30 ng/mL, sufficiency 30–150 ng/mL [25]. Estimated glomerular filtration rate (GFR) was calculated from serum creatinine using the CKD-EPI 2021 equation [26]. The Urinary Albumin-to-Creatinine Ratio (UACR) was determined from a spot urine specimen.

### 2.4. Statistical Analysis

All statistical analyses were performed using IBM SPSS Statistics (version 17.0.1) and GraphPad Prism (version 10). Normality was assessed using the Kolmogorov–Smirnov test; since the majority of continuous variables violated the normality assumption, non-parametric tests were applied throughout. Non-normally distributed continuous variables are reported as median (interquartile range (IQR): 25th–75th percentile); categorical variables as frequency and percentage.

Between-group comparisons were performed using the Kruskal–Wallis pairwise test with Dunn’s post hoc correction; effect sizes were quantified using epsilon-squared (ε^2^). For individual pairwise contrasts, the rank-biserial correlation (r) was additionally reported as the effect size for each individual Mann–Whitney U contrast. Within-group comparisons used the Mann–Whitney U test; categorical variables were compared using the chi-square test. Spearman’s rank correlation was used to assess bivariate associations. Benjamini–Hochberg (BH) false discovery rate (FDR) correction was applied across all 53 correlation tests; correlations with q < 0.05 were considered statistically significant. Raw *p*-values are reported alongside FDR-adjusted q-values in the tables. Age-adjusted partial Spearman correlations were additionally computed for all primary biomarker pairs to assess confounding by the substantial age difference between groups (38% overlap between control and T2DM age ranges); these are presented as sensitivity analyses in Supplementary Table S2. All other analyses are exploratory with no additional multiplicity correction.

To examine robustness of the primary between-group differences to confounding by age and BMI, median (50th percentile) quantile regression was performed for sLOX-1, 25(OH)D_3_, and UACR, with group membership as the predictor and age and BMI as covariates; results are expressed as adjusted ratios of medians with 95% confidence intervals. These analyses were performed using the quantreg package in R (version 4.3).

Binary logistic regression was performed with five pre-specified predictors: age, waist circumference, 25(OH)D_3_, sLOX-1, and log(UACR), yielding an events per variable (EPV) of 5.4. BMI was excluded owing to high collinearity with waist circumference (Spearman r = 0.870); sex was excluded to preserve EPV; HbA1c was excluded due to complete separation between groups. UACR was log-transformed prior to inclusion owing to extreme right skewness (skewness = 7.39). Influential observations were assessed using Cook’s distance (threshold: 4/*n* = 0.050). Firth’s penalized logistic regression was considered but could not be implemented in the available software. Model fit was evaluated using Akaike Information Criterion (AIC) and McFadden’s pseudo-R^2^. All regression estimates are exploratory given EPV < 10.

Multicollinearity among predictors was assessed using the variance inflation factor (VIF; threshold < 10) and the condition number of the predictor matrix (threshold < 30); results are reported. Where Cook’s distance identified influential observations (threshold: 4/*n* = 0.050), a sensitivity analysis was performed by refitting Model 1 after excluding those observations, and results were compared with the primary analysis. As an additional sensitivity analysis, Model 1A was fitted with the reference category restricted to normoglycaemic controls only (*n* = 53), to assess whether the associations identified in Model 1 reflected a generalised contrast with non-diabetic states or were driven by the T2DM–prediabetic contrast specifically. The incremental discriminative value of the three biomarkers (25(OH)D_3_, log(UACR), sLOX-1) beyond conventional covariates (age, waist circumference) was assessed using the likelihood-ratio test comparing a covariate-only model with the full five-predictor model. Discrimination was quantified using the area under the receiver operating characteristic curve (AUC), with 95% confidence intervals derived using the DeLong method. Bootstrap internal validation (B = 1000 iterations, sampling with replacement) was used to estimate optimism in the apparent AUC; the optimism-corrected AUC was calculated as the apparent AUC minus the mean bootstrap optimism.

An a priori power analysis was conducted using G*Power 3.1 (one-way ANOVA reference design, Cohen’s f = 0.35, α = 0.05, power = 80%), with serum sLOX-1 as the primary outcome, yielding a minimum of 28 participants per group (total *n* = 84). The enrolled sample of 80 approximated this requirement. This calculation applies to the primary three-group comparison only; regression and microvascular subgroup analyses are underpowered by design and are designated as exploratory throughout.

## 3. Results

### 3.1. Demographic Characteristics

The study comprised 80 participants, of whom 18 were male (22.5%) and 62 were female (77.5%). The overall age distribution differed significantly among the three groups (H = 32.58, *p* < 0.0001), with a large effect size (ε^2^ = 0.397, Cohen’s f = 0.812). Post hoc pairwise comparisons revealed that the control group was significantly younger than both the prediabetic group (rank-biserial r = 0.707, *p* < 0.0001) and the T2DM group (rank-biserial r = 0.873, *p* < 0.0001), whereas no significant age difference was observed between the prediabetic and T2DM groups (r = −0.016, *p* = 0.924). The median age of the control group was 38.0 years (IQR: 35.0–43.3), which was significantly lower than that of the prediabetic group [55.0 years (IQR: 44.0–63.0); *p* < 0.0001] and T2DM group [51.0 years (IQR: 47.0–59.0); *p* < 0.0001]. Sex distribution was comparable across all three groups (χ^2^(2) = 0.3423, *p* = 0.8427). The age overlap between control and T2DM groups was limited: only 10 of 26 controls (38%) fell within the T2DM age range (40–69 years). BMI overlap was somewhat greater, with 16 of 26 controls falling within the T2DM BMI range (22.9–65.4 kg/m^2^). These distributional differences should be considered when interpreting all between-group comparisons.

### 3.2. Anthropometric and Biochemical Parameters

Anthropometric and laboratory parameters are summarized in Table 1. BMI and waist circumference were significantly higher in both the prediabetic and T2DM groups compared to controls (*p* < 0.0001 for all), with no significant difference between Groups 2 and 3 (*p* > 0.9999).

Fasting glucose differed significantly across all three groups (H = 54.87, *p* < 0.0001, ε^2^ = 0.687). The prediabetic group showed moderately elevated fasting glucose compared to controls (*p* = 0.0183), while T2DM patients had markedly higher values than both controls and prediabetic individuals (*p* < 0.0001 for both). Similarly, HbA1c was significantly higher at each successive glycemic stage (H = 70.13, *p* < 0.0001, ε^2^ = 0.885), with all pairwise comparisons reaching statistical significance (Control vs. Prediabetic: *p* = 0.0001; Control vs. T2DM and Prediabetic vs. T2DM: *p* < 0.0001).

Among lipid parameters, total cholesterol differed significantly across groups (H = 12.82, *p* = 0.0016, ε^2^ = 0.140) and was significantly elevated in the prediabetic group compared to controls (*p* = 0.0010), whereas no significant difference was observed between the T2DM and control groups (*p* = 0.2106). Triglyceride levels differed significantly across groups (H = 28.18, *p* < 0.0001, ε^2^ = 0.340) and were significantly higher in both the prediabetic (*p* = 0.0020) and T2DM (*p* < 0.0001) groups relative to controls, with no significant difference between Groups 2 and 3 (*p* = 0.1901). LDL-cholesterol differed significantly across groups (H = 13.88, *p* = 0.0010, ε^2^ = 0.154) and was significantly higher in the prediabetic group than in both controls (*p* = 0.0011) and T2DM patients (*p* = 0.0215), while T2DM and control groups did not differ significantly (*p* > 0.9999). HDL-cholesterol differed significantly across groups (H = 9.76, *p* = 0.0076, ε^2^ = 0.101) and was significantly lower in the T2DM group compared to controls (*p* = 0.0054), with no significant difference between the prediabetic and control groups (*p* = 0.4610).

### 3.3. Serum sLOX-1, 25(OH)D_3_, and UACR Levels Across Study Groups

**Figure 1:**
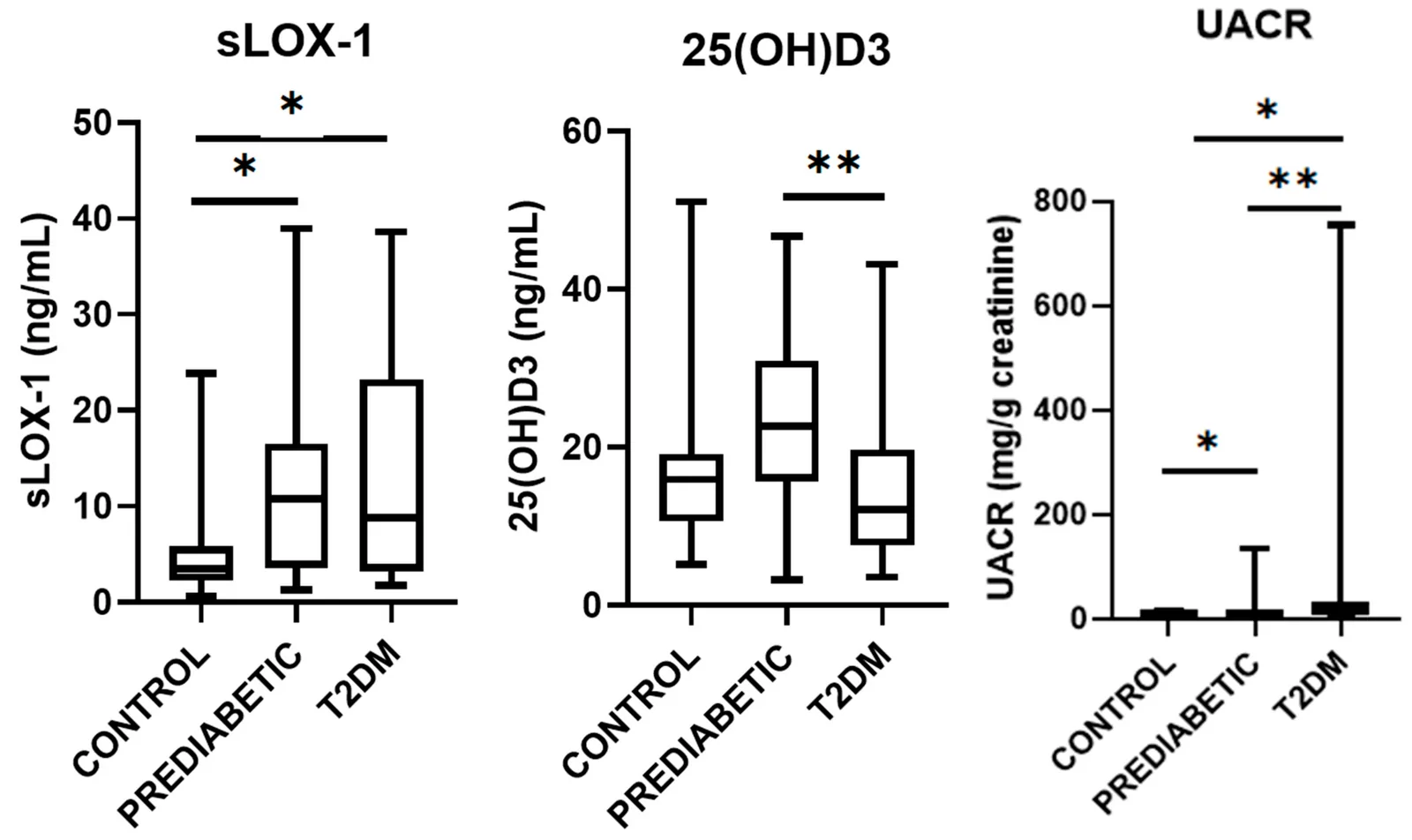
Box plots of serum sLOX-1 concentrations (ng/mL), 25(OH)D_3_ concentrations (ng/mL) and UACR (mg/g creatinine) across study groups. Horizontal lines within boxes indicate median values; box boundaries represent the 25th and 75th percentiles; whiskers extend to minimum and maximum values. Statistical comparisons were performed using the Kruskal–Wallis test with Dunn’s post hoc correction. * *p* < 0.05 vs. Group 1 (Control); ** *p* < 0.01 vs. Group 2 (Prediabetic). sLOX-1: soluble lectin-like oxidized low-density lipoprotein receptor-1; 25(OH)D_3_: 25-hydroxyvitamin D_3_; UACR: urinary albumin-to-creatinine ratio; T2DM: type 2 diabetes mellitus.

Serum sLOX-1 levels were significantly elevated in both the prediabetic [median 10.75 (IQR: 3.57–16.58) ng/mL; *p* = 0.0397] and T2DM [8.83 (3.21–23.26) ng/mL; *p* = 0.0209] groups compared to controls [3.50 (2.25–5.85) ng/mL] (Kruskal–Wallis H = 8.924, *p* = 0.0115; ε^2^ = 0.090, medium effect). No significant difference in sLOX-1 levels was observed between the prediabetic and T2DM groups (*p* > 0.9999). These findings are illustrated in Figure 1.

The vitamin D deficiency (<20 ng/mL) was present in 20 of 26 controls (77%), 10 of 27 prediabetic individuals (37%), and 21 of 27 T2DM patients (78%). Severe deficiency (<10 ng/mL) was particularly prevalent in the T2DM group (12/27, 44%) compared with controls (3/26, 12%) and prediabetic individuals (4/27, 15%).

Serum 25(OH)D_3_ levels differed significantly across groups (H = 11.25, *p* = 0.0036, ε^2^ = 0.117, medium effect) (Figure 1). Post hoc analysis revealed that 25(OH)D_3_ was significantly lower in the T2DM group [12.11 (7.59–19.74) ng/mL] than in the prediabetic group [22.69 (15.69–30.97) ng/mL; *p* = 0.0024], whereas neither the prediabetic nor the T2DM group differed significantly from controls [15.93 (10.64–19.15) ng/mL; *p* = 0.4009 and *p* = 0.2591, respectively].

UACR increased in a stepwise manner across the glycemic spectrum (H = 27.29, *p* < 0.0001, ε^2^ = 0.328, large effect) (Figure 1). UACR was significantly higher in the prediabetic group [7.47 (4.27–16.91) mg/g creatinine; *p* = 0.0331] and T2DM group [18.15 (8.15–34.29) mg/g creatinine; *p* < 0.0001] than in controls [4.99 (4.00–5.42) mg/g creatinine], and was additionally significantly higher in the T2DM group than in the prediabetic group (*p* = 0.002), indicating a stepwise increase in urinary albumin excretion across glycemic stages. All three group medians, however, remained below the clinical nephropathy threshold of 30 mg/g creatinine (Control: 4.99 mg/g; Prediabetic: 7.47 mg/g; T2DM: 18.15 mg/g), and these findings should not be interpreted as evidence of overt albuminuria or confirmed renal injury at the group level.

To examine robustness to confounding, quantile regression was performed adjusting for age and BMI (Table S1). After adjustment, the elevation of sLOX-1 in prediabetic individuals relative to controls remained statistically significant (adjusted ratio of medians = 2.91, 95% CI: 1.05–8.07, *p* = 0.040), suggesting that this difference is not fully explained by the age and BMI differences between groups. In contrast, the sLOX-1 elevation in T2DM relative to controls was substantially attenuated and no longer significant after adjustment (adjusted ratio = 1.81, 95% CI: 0.63–5.20, *p* = 0.265). For 25(OH)D_3_, the significantly lower concentrations in the T2DM group relative to controls survived age and BMI adjustment (adjusted ratio = 0.59, 95% CI: 0.35–0.97, *p* = 0.039), whereas the prediabetic versus control difference was non-significant after adjustment (ratio = 1.39, 95% CI: 0.85–2.27, *p* = 0.185). For UACR, the T2DM versus control difference remained highly significant after adjustment (adjusted ratio = 3.81, 95% CI: 1.90–7.67, *p* = 0.001), whereas the prediabetic versus control difference was borderline (adjusted ratio = 1.88, 95% CI: 0.96–3.69, *p* = 0.067). Statistical adjustment reduced but did not fully resolve between-group differences given the limited covariate overlap, particularly for the T2DM and control age ranges.

### 3.4. Correlation Analyses

Spearman’s rank correlation analysis was performed across all 80 participants. Benjamini–Hochberg (BH) false discovery rate (FDR) correction was applied across all 53 Spearman rank correlation tests performed in Table 2 and Table 3; correlations with a BH-adjusted q-value < 0.05 were considered statistically significant after correction for multiple testing (Table S2).

BMI and waist circumference were significantly and positively correlated with fasting glucose (r = 0.487, q < 0.001 and r = 0.491, q < 0.001, respectively) and triglycerides (r = 0.425, q < 0.001 and r = 0.451, q < 0.001, respectively), and negatively correlated with HDL-cholesterol (r = −0.299, q = 0.025 and r = −0.294, q = 0.026, respectively).

Fasting glucose was positively correlated with triglycerides (r = 0.536, q < 0.001) and UACR (r = 0.485, q < 0.001), and negatively correlated with HDL-cholesterol (r = −0.431, q < 0.001). Triglycerides were negatively correlated with HDL-cholesterol (r = −0.491, q < 0.001) and positively correlated with total cholesterol (r = 0.310, q = 0.019).

Regarding the primary biomarkers of interest, sLOX-1 showed a positive association with triglycerides (r = 0.270, *p* = 0.015) that did not survive FDR correction (q = 0.050). Similarly, the positive association between 25(OH)D_3_ and HDL-cholesterol (r = 0.273, *p* = 0.015) did not survive FDR correction (q = 0.050). No FDR-significant correlation was observed between sLOX-1 and 25(OH)D_3_ in the overall cohort.

Spearman’s rank correlation analysis was conducted to examine associations between the primary biomarkers (sLOX-1 and 25(OH)D_3_), UACR, waist circumference, and HbA1c across the total cohort, the prediabetic subgroup, and the T2DM subgroup. Results are summarized in Table 3.

After FDR correction, sLOX-1 showed significant positive associations with waist circumference (r = 0.346, q = 0.007) and HbA1c (r = 0.296, q = 0.025) in the unadjusted analysis. However, both associations were attenuated and no longer statistically significant after partial correlation analysis adjusting for age (waist circumference: r\u209A = 0.221, q = 0.111; HbA1c: r\u209A = 0.119, q = 0.387), indicating that the observed associations were substantially confounded by the age difference between groups. sLOX-1 did not correlate significantly with 25(OH)D_3_ (r = 0.054, *p* = 0.633) or UACR (r = 0.021, *p* = 0.851) in either the unadjusted or age-adjusted analysis. Serum 25(OH)D_3_ showed no significant correlations with waist circumference (r = −0.094, *p* = 0.405), HbA1c (r = −0.180, *p* = 0.110), or UACR (r = −0.182, *p* = 0.106) in the total cohort. UACR was significantly and positively correlated with both waist circumference (r = 0.359, q = 0.006) and HbA1c (r = 0.555, q < 0.001). After age adjustment, the HbA1c association remained statistically significant r_p_ = 0.506, q < 0.001), whereas the waist circumference association was attenuated and did not meet the FDR threshold (r_p_ = 0.261, q = 0.058). In contrast, the association between 25(OH)D_3_ and HbA1c, which was non-significant in the unadjusted analysis (r = −0.180, *p* = 0.110), strengthened and became statistically significant after age adjustment (r\u209A = −0.387, q = 0.003), suggesting that lower vitamin D concentrations are independently associated with higher glycemic burden. The inverse association between UACR and 25(OH)D_3_ showed a non-significant trend after age adjustment (r_p_ = −0.248, q = 0.070). Age-adjusted associations between UACR and fasting glucose (r_p_ = 0.412, *p* = 0.0001) and triglycerides (r_p_ = 0.303, *p* = 0.006) also remained statistically significant; these results are reported in Supplementary Table S2.

In the T2DM subgroup analysis (*n* = 27), none of the examined correlations reached statistical significance. Similarly, no significant correlations were identified within the prediabetic subgroup (*n* = 27). Given the small subgroup sizes, these analyses were underpowered, and the absence of significant within-group associations is consistent with either a genuine lack of association within these subgroups or the confounding effect of the age and adiposity differences demonstrated in the total-cohort analyses; insufficient statistical power may also contribute but cannot be distinguished from these other explanations on the basis of the available data.

### 3.5. sLOX-1 and 25(OH)D_3_ According to Retinopathy or Elevated Albuminuria Status

Within the T2DM group (*n* = 27), retinopathy was present in 6 subjects (22.2%) and absent in 21 (77.8%). Data stratified by retinopathy status are presented in Table 4. Age did not differ significantly between patients with and without retinopathy [median 62.5 (IQR: 47.0–63.75) vs. 51.0 (47.0–56.0) years; *p* = 0.3192], whereas diabetes duration was significantly longer in the retinopathy group [13.0 (10.25–20.25) vs. 6.0 (4.0–10.0) years; *p* = 0.0174]. Serum sLOX-1 did not differ significantly between patients with and without retinopathy [5.63 (5.35–13.23) vs. 11.65 (3.21–23.26) ng/mL; *p* = 0.5402], nor did 25(OH)D_3_ levels [18.11 (10.18–20.17) vs. 10.09 (8.67–19.30) ng/mL; *p* = 0.6289].

Serum creatinine was available for all participants. Estimated GFR was within the normal range in all three groups (Control: median 114.1 mL/min/1.73 m^2^, IQR 103.3–118.0; Prediabetic: 96.4, IQR 86.5–105.6; T2DM: 106.9, IQR 93.8–113.7), and no participant had an eGFR below 60 mL/min/1.73 m^2^.

Elevated albuminuria (UACR ≥ 30 mg/g creatinine) was identified in 10 of the 27 T2DM patients (37.0%). Data stratified by albuminuria status are presented in Table 4.

Neither age nor diabetes duration differed significantly between patients with and without elevated albuminuria [age: 49.5 (42.75–60.5) vs. 52.0 (51.0–59.0) years, *p* = 0.3379; diabetes duration: 10.0 (9.25–10.75) vs. 6.0 (5.0–14.0) years, *p* = 0.2552]. sLOX-1 was numerically higher in the elevated albuminuria group [15.70 (5.39–21.80) vs. 7.52 (3.21–22.15) ng/mL] but not significantly so (*p* = 0.6513), and 25(OH)D_3_ was numerically lower [8.13 (6.91–20.17) vs. 12.25 (9.38–19.30) ng/mL; *p* = 0.4074]. Given the small and unequal subgroup sizes, these exploratory analyses were underpowered and permit no conclusion in either direction regarding an association between the biomarkers and microvascular complications.

### 3.6. Logistic Regression Analysis

Binary logistic regression was performed using five pre-specified predictors: age, waist circumference, 25(OH)D_3_, log(UACR), and sLOX-1. With 27 outcome events, this yields an EPV of 5.4, which remains below the recommended threshold of 10; all regression estimates are therefore treated as exploratory and hypothesis-generating. HbA1c was excluded from both models due to complete separation between groups (T2DM range 6.5–12.9%; control range 4.5–5.6%), and BMI was excluded given its high collinearity with waist circumference (Spearman r = 0.870). In the five-predictor model, all VIF values were below 2 and the condition number was 2.1, indicating no collinearity concern (Supplementary Table S3, Panel A). The cross-sectional design does not permit causal or temporal inference from these associations. Results are presented in Table 5.

Cook’s distance analysis identified seven influential observations in Model 1 (Cook’s D > 0.050). A sensitivity analysis excluding these observations showed that the 25(OH)D_3_ association remained significant (OR = 0.761, 95% CI: 0.616–0.940, *p* = 0.011). However, the log(UACR) odds ratio became extreme (OR > 700), indicating that this estimate is substantially sensitive to four observations with very high UACR values (>100 mg/g, maximum = 755.1 mg/g). This instability should be considered when interpreting the log(UACR) regression result.

In Model 1, controls and prediabetic individuals were combined as the reference category in order to maximise the effective sample size of the reference group given the small total sample (*n* = 80). This comparison addresses the question of whether T2DM is distinguished from any non-diabetic state after covariate adjustment. We acknowledge, however, that the control and prediabetic groups differ substantially in metabolic and vitamin D parameters. As a sensitivity analysis, Model 1A restricted the reference category to controls only (*n* = 53, 27 T2DM events, EPV = 5.4). In Model 1A, the 25(OH)D_3_ association was no longer statistically significant (OR = 1.008, 95% CI: 0.755–1.344, *p* = 0.959), whereas log(UACR) remained significant but with an extremely wide confidence interval (OR = 67.9, 95% CI: 1.14–4039, *p* = 0.043), likely reflecting severe overfitting in this reduced sample (McFadden R^2^ = 0.899). These results indicate that the 25(OH)D_3_ finding in Model 1 is substantially driven by the contrast between the T2DM and prediabetic groups rather than a generalised distinction from normoglycaemia, consistent with the prediabetic group having the highest median 25(OH)D_3_ concentrations in the cohort.

In Model 1 (T2DM vs. Control and Prediabetic combined; *n* = 80; EPV = 5.4; McFadden R^2^ = 0.402), serum 25(OH)D_3_ was independently associated with T2DM status after adjustment (OR = 0.922, 95% CI: 0.861–0.987, *p* = 0.020). Log(UACR) was also independently associated with T2DM status (OR = 4.014, 95% CI: 1.793–8.986, *p* = 0.001), though this estimate was sensitive to influential observations as described above. sLOX-1 was not significantly associated with T2DM status (OR = 1.058, 95% CI: 0.982–1.139, *p* = 0.136). Among the covariates, age was independently associated with the outcome (OR = 1.111, 95% CI: 1.036–1.192, *p* = 0.003), while waist circumference was not significant (OR = 0.997, *p* = 0.882).

In Model 2 (Prediabetic vs. Control; *n* = 53; EPV = 5.4; McFadden R^2^ = 0.583), none of the primary biomarkers reached statistical significance: 25(OH)D_3_ (OR = 1.068, 95% CI: 0.972–1.174, *p* = 0.169), log(UACR) (OR = 3.250, 95% CI: 0.917–11.516, *p* = 0.068), and sLOX-1 (OR = 1.018, 95% CI: 0.896–1.157, *p* = 0.778). Age was the only covariate independently associated with the outcome (OR = 1.159, 95% CI: 1.028–1.306, *p* = 0.016); waist circumference showed a borderline association (OR = 1.082, 95% CI: 0.993–1.178, *p* = 0.072).

To explore the incremental value of the biomarkers beyond conventional risk factors, a covariate-only model (age and waist circumference) was compared with the full model additionally including 25(OH)D_3_, log(UACR), and sLOX-1. Adding the three biomarkers improved model performance (likelihood-ratio χ^2^(3) = 22.6, *p* < 0.001), with the apparent area under the ROC curve increasing from 0.730 (covariate-only) to 0.899 (full model; 95% CI: 0.827–0.972) in the development sample. Bootstrap internal validation (B = 1000 iterations) estimated a mean optimism of 0.028, yielding an optimism-corrected AUC of 0.871. These figures represent in-sample performance estimates from the same small dataset used for model development and should not be interpreted as evidence of validated prognostic utility; external validation in an independent sample is required. sLOX-1 alone did not significantly improve model fit when added to the model containing age, waist circumference, 25(OH)D_3_, and log(UACR) (χ^2^(1) = 2.39, *p* = 0.122, ΔAUC = 0.013), confirming the absence of independent incremental discrimination for sLOX-1 in this dataset.

## 4. Discussion

This prospective cross-sectional study examined the associations between serum sLOX-1 and 25(OH)D_3_ levels, microvascular complications, metabolic parameters, and glycemic control in prediabetic and T2DM individuals. Importantly, while previous studies have examined sLOX-1 and vitamin D status in diabetic populations separately [13,15,16,17,18,23,28,29], this is one of the first studies, to our knowledge, to evaluate both biomarkers simultaneously across the prediabetes and T2DM spectrum. The principal findings can be summarised as follows: in unadjusted analyses, serum sLOX-1 was significantly elevated in both prediabetic and T2DM individuals relative to normoglycemic controls. After adjustment for age and BMI by quantile regression, the sLOX-1 elevation in prediabetic individuals persisted, whereas the elevation in T2DM relative to controls was substantially attenuated and no longer statistically significant, consistent with the large age and BMI differences between groups. Serum 25(OH)D_3_ levels were significantly lower in the T2DM group than in prediabetic individuals, with a high prevalence of vitamin D deficiency observed among T2DM patients. UACR increased in a stepwise fashion across the glycemic spectrum and was independently associated with T2DM status in logistic regression, as was 25(OH)D_3_, although the latter association was not robust to changes in reference-group composition. Given the cross-sectional design, these associations do not permit causal or temporal inference. In unadjusted analyses, sLOX-1 correlated positively with waist circumference and HbA1c across the total cohort; however, both associations were no longer significant after age adjustment and should be interpreted as hypothesis-generating only. In the T2DM subgroup, neither sLOX-1 nor 25(OH)D_3_ differed significantly between patients with and without microvascular complications, given the very small subgroup size. These exploratory analyses were underpowered and permit no conclusion in either direction. Notably, sLOX-1 and 25(OH)D_3_ were not significantly correlated in this cohort.

LOX-1 is a type II transmembrane glycoprotein that is predominantly expressed on vascular endothelial cells, macrophages and smooth muscle cells. When it binds to oxidised LDL (oxLDL), LOX-1 triggers a series of events that promote inflammation and atherosclerosis, including the activation of NF-κB, the generation of ROS, the upregulation of adhesion molecules, and endothelial apoptosis [11,28]. The soluble form of LOX-1, which is produced by the proteolytic cleavage of the membrane-bound receptor, has been associated with endothelial activation and oxidative vascular injury [9,10,11].

In the present study, serum sLOX-1 levels were higher in both the prediabetic and T2DM groups compared with normoglycemic controls in unadjusted analyses. After adjustment for age and BMI, the elevation in the prediabetic group persisted, whereas the difference between the T2DM and control groups was attenuated and no longer statistically significant, consistent with the substantial age and BMI differences between groups. The persistent elevation at the prediabetic stage is consistent with the presence of endothelial activation early in dysglycemia and with previous studies that have also reported higher sLOX-1 levels in prediabetes and uncomplicated T2DM [4,23]. At the cellular level, Ling Li et al. [29] demonstrated that high glucose concentrations dose-dependently increase LOX-1 mRNA and protein expression in human aortic endothelial cells via ROS-mediated PKC/MAPK-NF-κB signaling. Although sLOX-1 was associated with waist circumference and HbA1c in the unadjusted analysis, these associations were no longer significant after adjusting for age. Because the control group was substantially younger than the dysglycemic groups (median 38 vs. 51–55 years), these pooled correlations were confounded by age and should be interpreted as hypothesis-generating rather than as evidence of an age-independent association; this attenuation does not, however, exclude a biological relationship that a larger, age-matched study might detect. The positive association with triglycerides did not survive FDR correction and is not further interpreted. Elevated sLOX-1 in metabolic disease has also been reported by Civelek et al. [12], who found positive correlations between sLOX-1 and oxLDL, endothelin-1, and BMI in metabolic syndrome patients, and by Stankova et al. [30], who identified elevated sLOX-1 as a marker of coronary artery disease risk within metabolic syndrome populations. Our study extends these observations to the prediabetic stage.

Chronic glycemic exposure and atherogenic dyslipidemia may act together to increase LOX-1 upregulation and shedding in individuals with glucose dysregulation. Experimental work has reported that LOX-1 activation promotes oxidative stress and that vitamin D may attenuate this in cellular models [28,31,32]. No redox markers, endothelial function, or pathway activity were measured, and sLOX-1 and 25(OH)D_3_ were not significantly correlated in our cohort. The preceding pathways are therefore presented as biological context from the existing literature rather than as findings established by the present study. Circulating sLOX-1 may nonetheless warrant direct investigation alongside established oxidative stress markers in future studies. We observed no significant difference in sLOX-1 levels between the prediabetic and T2DM groups. This should be interpreted as the absence of a statistically significant difference between these two groups rather than as evidence that sLOX-1 does not change with glycemic status.

Diabetic microvascular complications, including nephropathy and retinopathy, are associated with the cumulative effects of hyperglycemia-induced endothelial dysfunction, oxidative stress and activation of the inflammatory pathway on small blood vessels [3]. We acknowledge that microvascular and macrovascular complications are distinct pathophysiological entities that should not be conflated. The present study focused specifically on microvascular endpoints, namely elevated albuminuria and retinopathy. The well-established associations of sLOX-1 with macrovascular disease were not evaluated in this cohort as they fall outside the scope of the present analysis. In our study, sLOX-1 levels did not differ significantly between patients with T2DM with or without elevated albuminuria or retinopathy. Because these subgroups were very small, the analyses were underpowered and should be regarded as exploratory only. They do not support any conclusion, in either direction, regarding an association between these biomarkers and microvascular complications. Notably, patients with retinopathy had significantly longer diabetes duration than those without, consistent with the established role of disease chronicity in microvascular damage, although the small retinopathy subgroup (*n* = 6) precludes any firm conclusion; age did not differ significantly between the two groups. The positive correlation between HbA1c and UACR observed across the entire cohort is consistent with the recognised association between glycemic burden and glomerular injury [3], although the cross-sectional design of the present study cannot establish causality. Furthermore, in binary logistic regression analysis, log(UACR) was independently associated with T2DM status after adjustment for age and waist circumference, consistent with its recognised role as a marker of glomerular injury. As shown in the Results, adding the biomarkers improved discrimination beyond covariates; however, HbA1c could not be evaluated as an incremental comparator because it was excluded owing to complete separation and is effectively collinear with the diagnostic outcome. These preliminary, hypothesis-generating findings should be confirmed in larger prospective cohorts using formal reclassification methods (NRI/IDI).

Another important finding of our study was the high prevalence of vitamin D deficiency among T2DM patients (78%; severe deficiency in 44%). Vitamin D has been consistently linked by epidemiological evidence to insulin resistance, impaired glycemic control and an increased risk of incident T2DM in individuals with prediabetes [15,33]. Importantly, Chen et al. [34] reported a sex-dependent association between 25(OH)D and insulin resistance, with an inverse and independent relationship observed specifically in vitamin D-deficient women. As 77.5% of our cohort were female, this context is directly relevant and suggests that sex-stratified analyses should be a priority in future work. In the present study, 25(OH)D_3_ levels were significantly lower in the T2DM group than in the prediabetes group. Vitamin D deficiency (<20 ng/mL) has frequently been reported among T2DM patients [35,36], attributed to reduced sunlight exposure, adipose tissue sequestration and impaired renal 1α-hydroxylase activity [14,15]. In our total cohort, 25(OH)D_3_ showed a borderline inverse association with fasting glucose in unadjusted analysis that did not survive correction for multiple comparisons. Notably, however, the inverse association between 25(OH)D_3_ and HbA1c, which was not significant in unadjusted analysis, strengthened and became significant after adjustment for age (r_p_ = −0.387, q = 0.003), suggesting that lower vitamin D concentrations are associated with higher glycemic burden independently of age. Nonetheless, binary logistic regression identified 25(OH)D_3_ as independently associated with T2DM status after adjustment for age and waist circumference, although this association was not robust to changes in reference-group composition and was substantially driven by the contrast between the T2DM and prediabetic groups. Prior studies have suggested vitamin D may influence nephropathy and retinopathy risk through VDR-mediated mechanisms [37,38], though these pathways were not evaluated in the present study. Furthermore, Tsuprykov et al. [39] have highlighted that total 25(OH)D_3_ may not fully capture biologically active vitamin D, and that free 25(OH)D may be a more relevant metric, particularly in patients with renal impairment or altered vitamin D-binding protein levels [40]. Future studies should therefore measure free 25(OH)D_3_ alongside total 25(OH)D_3_ to better characterise VDR-activating capacity across the glycemic spectrum. Li et al. [41] have further suggested that optimal 25(OH)D target ranges for endothelial protection and metabolic outcomes may lie between 30 and 50 ng/mL, a threshold not reached by any group in the present study, underscoring the degree of vitamin D deficiency in this cohort.

The unexpected pattern of 25(OH)D_3_, higher in prediabetic individuals than in both controls and T2DM patients, warrants critical discussion and weakens a simple glycemic-gradient interpretation of vitamin D status. Several factors may explain this finding. First, the control group consisted of hospital employees who may have had limited daytime sunlight exposure owing to occupational indoor working patterns, though this was not directly measured, which is a recognised independent contributor to suboptimal vitamin D status independent of glycemic status. Second, the control group was significantly younger (median 38 years) than the prediabetic group (median 55 years). Although younger age is typically associated with greater outdoor activity, occupational indoor confinement may override this. Third, the high national background prevalence of vitamin D deficiency in Turkish women, ranging from 50.5% to 78.7% across major cities [42], means that all groups started from a low population baseline, attenuating the expected inter-group gradient. Taken together, these factors suggest that 25(OH)D_3_ differences across groups may reflect not only glycemic status but also occupational, age-related, and population-level determinants, and must be interpreted accordingly. A meta-analysis has reported that vitamin D supplementation reduces the risk of incident T2DM in individuals with prediabetes [33], and the 2024 ADA Standards of Care discuss the potential role of vitamin D in this setting while noting that further evidence is required [43].

The present study adds to the limited literature by evaluating sLOX-1 and 25(OH)D_3_ simultaneously across the dysglycemic spectrum. Our data show that sLOX-1 is elevated in prediabetes and T2DM relative to controls, while 25(OH)D_3_ is significantly lower only in the T2DM group compared to prediabetic individuals and does not show a progressive decline across the glycemic spectrum. These two biomarkers were not significantly correlated with one another in any analysis. We therefore describe this pattern as a descriptive observation rather than evidence of a shared mechanistic pathway. Experimental studies have reported that vitamin D can modulate NF-κB signalling and that LOX-1 activation promotes NF-κB–driven inflammation [11,23,44], but whether these pathways interact in the context of dysglycemia cannot be determined from the present cross-sectional data. Whether targeted vitamin D supplementation in prediabetic or T2DM patients with documented vitamin D deficiency leads to a measurable reduction in circulating sLOX-1 remains a hypothesis that should be tested prospectively.

The present study has several limitations that must be acknowledged. First, the cross-sectional design precludes causal or temporal inference; it is not possible to determine whether the observed biomarker differences preceded or followed the development of glycemic or renal abnormalities. Second, the control group was recruited from hospital staff and was substantially younger and leaner than patient groups, with only 38% of controls falling within the T2DM age range. These differences were only partially addressable through quantile regression adjustment, given limited covariate overlap; caution is therefore warranted in attributing between-group biomarker differences to glycemic status alone. Third, the overall sample size was modest, and the microvascular subgroups were very small; all microvascular analyses are strictly descriptive and exploratory, and no inferential conclusions can be drawn from them. Retinopathy was classified dichotomously without a standardised grading system, and elevated albuminuria was defined on a single spot-urine UACR measurement rather than repeated confirmatory samples, which does not exclude transient albuminuria. The stepwise UACR increase across groups should be interpreted as a gradient in urinary albumin excretion rather than confirmed renal injury, as all group medians remained below the albuminuria threshold of 30 mg/g creatinine. Fourth, the logistic regression models had an EPV of 5.4, well below the recommended minimum of 10, raising concerns about overfitting; Firth’s penalized logistic regression could not be implemented in the available software and is recommended for future analyses. The log(UACR) estimate in Model 1 was sensitive to observations with extreme values, and the 25(OH)D_3_ finding was not robust when the reference category was restricted to controls only, further limiting the interpretation of regression results. Fifth, direct measurements of oxLDL, HOMA-IR, endothelial function markers, VDR activity, and NF-κB pathway activity were not obtained, which limits mechanistic interpretation. Data on concomitant medications (notably statin use) and smoking were unavailable. All samples were collected within a single three-month winter window (November 2017–January 2018), which limited seasonal variability in 25(OH)D_3_ concentrations. Because no meaningful temporal variation in collection date existed across participants within this narrow period, a date-based sensitivity analysis was not feasible. Individual sunlight exposure was not systematically captured. Finally, the predominantly female cohort (77.5%) and single-centre design limit generalisability.

Future studies should adopt prospective longitudinal designs with age- and BMI-matched recruitment across glycemic groups to permit causal inference and to disentangle the contributions of glycemic status, adiposity, and ageing to sLOX-1 and 25(OH)D_3_ levels. Larger multicentre cohorts with adequate EPV would enable robust adjusted modelling, ideally with Firth’s penalized estimation, and permit phenotype-specific subgroup analyses by sex, diabetic endotype, and pharmacological treatment status. Direct measurement of oxLDL, free 25(OH)D_3_, VDR activity, and endothelial oxidative stress markers alongside sLOX-1 would be necessary to clarify whether these biomarkers are mechanistically linked. Interventional studies examining whether vitamin D supplementation in prediabetic or T2DM individuals with documented deficiency leads to measurable reductions in circulating sLOX-1 would provide the most direct test of this hypothesis.

## 5. Conclusions

This study shows that serum sLOX-1 is higher at the prediabetic stage than in normoglycemic controls, a difference that persisted after adjustment for age and BMI; the elevation in the T2DM group, by contrast, was attenuated and no longer significant after adjustment. This prediabetic-stage elevation is consistent with endothelial activation early in dysglycemia. Its associations with central adiposity and glycemic burden did not persist after adjustment for age, indicating that they were largely confounded by the age difference between groups. Serum 25(OH)D_3_ was lower in T2DM than in prediabetic individuals and was associated with T2DM status after adjustment in cross-sectional logistic regression, although this association was not robust to changes in reference-group composition and should therefore be interpreted with caution. A high prevalence of vitamin D deficiency was observed among T2DM patients. However, sLOX-1 and 25(OH)D_3_ were not significantly correlated in this cohort, and no direct measures of endothelial function or vitamin D receptor signalling were obtained. Larger prospective studies with age-matched recruitment, incorporating direct measurement of oxLDL, endothelial function, and inflammatory markers, together with vitamin D supplementation interventions, are needed to clarify whether these two biomarkers are mechanistically linked and whether correction of vitamin D deficiency leads to measurable improvements in endothelial function in high-risk dysglycemic individuals.

## Figures and Tables

**Table 1:** Anthropometric and Biochemical Parameters Across Study Groups.

Variable	Group 1 Control (*n* = 26)	Group 2 Prediabetic (*n* = 27)	Group 3 T2DM (*n* = 27)	*p* Value	ε^2^
BMI (kg/m^2^), Median (IQR)	24.2 (21.3–25.8)	31.4 (27.5–35.9) ^a^	29.4 (27.9–35.1) ^a^	<0.0001	0.327
Waist circumference (cm), Median (IQR)	80.5 (73.8–94.3)	106.0 (97.0–122.0) ^a^	104.0 (98.0–115.0) ^a^	<0.0001	0.364
Fasting glucose (mg/dL), Median (IQR)	91.5 (85.5–96.0)	102.0 (94.0–110.0) ^b^	185.0 (144.0–223.0) ^a,c^	<0.0001	0.687
HbA1c (%), Median (IQR)	5.2 (5.1–5.5)	6.0 (5.9–6.2) ^a^	8.3 (7.5–9.3) ^a,c^	<0.0001	0.885
Total cholesterol (mg/dL), Median (IQR)	188.0 (171.0–202.3)	226.0 (201.0–246.0) ^a^	198.0 (174.0–231.0)	0.0016	0.140
Triglycerides (mg/dL), Median (IQR)	85.0 (64.8–105.5)	116.0 (88.0–183.0) ^a^	158.0 (115.0–245.0) ^a^	<0.0001	0.340
HDL-cholesterol (mg/dL), Median (IQR)	57.0 (53.0–61.5)	49.0 (44.0–63.0)	48.0 (42.0–53.0) ^a^	0.0076	0.101
LDL-cholesterol (mg/dL), Median (IQR)	109.5 (97.0–130.5)	145.0 (119.0–161.0) ^a^	113.0 (97.0–140.0) ^c^	0.0010	0.154

Data are presented as median (IQR: 25th–75th percentile). All comparisons performed using the Kruskal–Wallis test with Dunn’s post hoc correction. ^a^ *p* < 0.01 vs. Group 1 (Control); ^b^ *p* < 0.05 vs. Group 1 (Control); ^c^ *p* < 0.05 vs. Group 2 (Prediabetic). BMI: body mass index; HDL: high-density lipoprotein; IQR: interquartile range; LDL: low-density lipoprotein; T2DM: type 2 diabetes mellitus. ε^2^: epsilon-squared effect size for Kruskal–Wallis test; values of 0.01, 0.06, and 0.14 correspond approximately to small, medium, and large effects, respectively [27].

**Table 2:** Spearman Correlation Coefficients Between Anthropometric and Biochemical Parameters (All Participants Combined, *n* = 80).

Variable	Glucose			Triglycerides			Total Cholesterol			HDL		
	r	*p*-Value	q (FDR)	r	*p*-Value	q (FDR)	r	*p*-Value	q (FDR)	r	*p*-Value	q (FDR)
BMI	0.487	<0.001	**<0.001**	0.425	<0.001	**<0.001**	0.094	0.406	0.652	−0.298	0.008	**0.025**
Waist circumference	0.491	<0.001	**<0.001**	0.451	<0.001	**<0.001**	0.087	0.443	0.655	−0.294	0.020	**0.025**
Triglycerides	0.536	<0.001	**<0.001**	–	–	–	0.310	0.006	**0.019**	−0.491	<0.001	**<0.001**
HDL	−0.431	<0.001	**<0.001**	−0.491	<0.001	**<0.001**	0.247	0.027	0.068	–	–	–
sLOX-1	0.202	0.073	0.154	0.270	0.015	0.050	−0.044	0.699	0.837	−0.173	0.124	0.219
25(OH)D_3_	−0.220	0.050	0.121	0.019	0.868	0.902	0.187	0.097	0.190	0.273	0.015	0.050
UACR	0.485	<0.001	**<0.001**	0.363	0.001	**0.004**	0.257	0.021	0.057	−0.084	0.457	0.655

Values are Spearman’s rank correlation coefficient (r). *p*: raw *p*-value. q (FDR): Benjamini–Hochberg false discovery rate-adjusted *p*-value. Bold values indicate FDR-significant correlations (q < 0.05). BMI: body mass index; UACR: urinary albumin-to-creatinine ratio; sLOX-1: soluble lectin-like oxidized low-density lipoprotein receptor-1; 25(OH)D_3_: 25-hydroxyvitamin D_3_; HDL: high-density lipoprotein. –: self-comparison or duplicate pair.

**Table 3:** Spearman Correlation Coefficients Between Waist Circumference, HbA1c, sLOX-1, 25(OH)D_3_, and UACR Levels (Total: All Participants, Prediabetic or T2DM Subgroup).

Variable	Waist Circumference			HbA1c			sLOX-1			25(OH)D_3_			UACR		
	r	*p*-Value	q (FDR)	r	*p*-Value	q (FDR)	r	*p*-Value	q (FDR)	r	*p*-Value	q (FDR)	r	*p*-Value	q (FDR)
Total Cohort (*n =* 80)															
sLOX-1	0.346	0.002	**0.007**	0.296	0.008	**0.025**	–	–	–	0.054	0.633	0.818	0.021	0.851	0.902
25(OH)D_3_	−0.094	0.405	0.652	−0.180	0.110	0.201	0.054	0.633	0.818	–	–	–	−0.182	0.106	0.200
UACR	0.359	0.001	**0.005**	0.555	<0.001	**<0.001**	0.021	0.851	0.902	−0.182	0.106	0.200	–	–	–
Prediabetic subgroup (*n* = 27)															
sLOX-1	0.198	0.322	0.551	0.127	0.529	0.701	–	–	–	−0.090	0.655	0.827	−0.361	0.064	0.148
25(OH)D_3_	−0.151	0.452	0.655	0.080	0.693	0.837	−0.090	0.655	0.827	–	–	–	0.018	0.930	0.930
UACR	0.153	0.446	0.655	−0.075	0.711	0.837	−0.361	0.064	0.148	0.018	0.930	0.930	–	–	–
T2DM subgroup (*n* = 27)															
sLOX-1	0.023	0.910	0.928	0.138	0.493	0.688	–	–	–	0.039	0.845	0.902	−0.127	0.527	0.701
25(OH)D_3_	−0.056	0.782	0.895	−0.335	0.088	0.179	0.039	0.845	0.902	–	–	–	−0.355	0.069	0.152
UACR	0.053	0.794	0.895	0.035	0.863	0.902	−0.127	0.527	0.701	−0.355	0.069	0.152	–	–	–

Values are Spearman’s rank correlation coefficient (r). *p*: raw *p*-value. q (FDR): Benjamini–Hochberg false discovery rate-adjusted *p*-value. Bold values indicate FDR-significant correlations (q < 0.05). UACR: urinary albumin-to-creatinine ratio; sLOX-1: soluble lectin-like oxidized low-density lipoprotein receptor-1; 25(OH)D_3_: 25-hydroxyvitamin D_3_; HbA1c: glycated haemoglobin; T2DM: type 2 diabetes mellitus. –: self-comparison.

**Table 4:** sLOX-1 and 25(OH)D_3_ According to Retinopathy or Albuminuria Status in the T2DM Group.

Variable	Retinopathy Present (*n* = 6)	Retinopathy Absent (*n* = 21)	*p*-Value
Age (years), Median (IQR)	62.50 (47.00–63.75)	51.00 (47.00–56.00)	0.3192
Diabetes duration (years), Median (IQR)	13.0 (10.25–20.25)	6.0 (4.00–10.00)	**0.0174**
sLOX-1 (ng/mL), Median (IQR)	5.63 (5.35–13.23)	11.65 (3.21–23.26)	0.5402
25(OH)D_3_ (ng/mL), Median (IQR)	18.11 (10.18–20.17)	10.09 (8.67–19.30)	0.6289
**Variable**	**UACR ≥ 30** **(*n* = 10)**	**UACR < 30** **(*n* = 17)**	***p*-Value**
Age (years), Median (IQR)	49.50 (42.75–60.50)	52.00 (51.00–59.00)	0.3379
Diabetes duration (years), Median (IQR)	10.00 (9.25–10.75)	6.00 (5.00–14.00)	0.2552
sLOX-1 (ng/mL), Median (IQR)	15.70 (5.39–21.80)	7.52 (3.21–22.15)	0.6513
25(OH)D_3_ (ng/mL), Median (IQR)	8.13 (6.91–20.17)	12.25 (9.38–19.30)	0.4074

Data are presented as median (IQR: 25th–75th percentile). All comparisons performed using the Mann–Whitney U test (two-tailed). Bold values indicate statistically significant differences (*p* < 0.05). sLOX-1: soluble lectin-like oxidized low-density lipoprotein receptor-1; 25(OH)D_3_: 25-hydroxy vitamin D_3_; IQR: interquartile range; T2DM: type 2 diabetes mellitus.

**Table 5:** Binary Logistic Regression Analysis: Predictors of T2DM and Prediabetes Status.

Variable	Model 1: T2DM vs. Control + Prediabetic (*n* = 80, Events = 27, EPV = 5.4)			Model 2: Prediabetic vs. Control (*n* = 53, Events = 27, EPV = 5.4)		
	OR	95% CI	*p*-Value	OR	95% CI	*p*-Value
Covariates (pre-specified)						
Age (years)	1.111	1.036–1.192	**0.003**	1.159	1.028–1.306	**0.016**
Waist circumference (cm)	0.997	0.954–1.041	0.882	1.082	0.993–1.178	0.072
Primary predictors of interest						
25(OH)D_3_ (ng/mL)	0.922	0.861–0.987	**0.020**	1.068	0.972–1.174	0.169
log(UACR) †	4.014	1.793–8.986	**0.001**	3.250	0.917–11.516	0.068
sLOX-1 (ng/mL)	1.058	0.982–1.139	0.136	1.018	0.896–1.157	0.778
Model fit: Model 1; AIC = 73.2, McFadden R^2^ = 0.402, apparent AUC = 0.899 (95% CI: 0.827–0.972), optimism-corrected AUC = 0.871. Model 2; AIC = 42.7, McFadden R^2^ = 0.583.						

Bold values indicate statistical significance (*p* < 0.05). OR: odds ratio; CI: confidence interval. † UACR was log-transformed prior to analysis (raw UACR skewness = 7.39, maximum = 755.1 mg/g). The OR for log(UACR) represents the multiplicative change in odds per one-unit increase in log(UACR). Cook’s distance analysis identified seven influential observations in Model 1; sensitivity analysis excluding these observations showed that the 25(OH)D_3_ association remained significant (OR = 0.761, *p* = 0.011), whereas the log(UACR) estimate became extreme (OR > 700), indicating substantial sensitivity to observations with very high UACR values. EPV: events per variable; AIC: Akaike Information Criterion; OR: odds ratio; CI: confidence interval; UACR: urinary albumin-to-creatinine ratio; sLOX-1: soluble lectin-like oxidized low-density lipoprotein receptor-1; 25(OH)D_3_: 25-hydroxyvitamin D_3_; T2DM: type 2 diabetes mellitus; BMI: body mass index.

## Data Availability

The data presented in this study are available on request from the corresponding author. The data are not publicly available due to privacy restrictions related to patient information.

## Supplementary Materials

The following supporting information can be downloaded at: https://www.mdpi.com/article/10.3390/jcm15207989/s1, Table S1. Unadjusted and Age- and BMI-Adjusted Between-Group Comparisons for Primary Biomarkers (*n* = 80). Table S2. Unadjusted and Age-Adjusted Partial Spearman Rank Correlation Coefficients for Primary Biomarkers (*n* = 80). Table S3. Multicollinearity Assessment and Sensitivity Analyses for Logistic Regression Models (5-Predictor Specification).

## Abbreviations

The following abbreviations are used in this manuscript:

25(OH)D_3_	25-hydroxyvitamin D_3_
AGE	advanced glycation end-products
AIC	Akaike Information Criterion
ADA	American Diabetes Association
BH_4_	tetrahydrobiopterin
BMI	body mass index
CI	confidence interval
CYP27B1	cytochrome P450 family 27 subfamily B member 1
DM	diabetes mellitus
ELISA	enzyme-linked immunosorbent assay
eNOS	endothelial nitric oxide synthase
HbA1c	glycated haemoglobin
HDL	high-density lipoprotein
HPLC	high-performance liquid chromatography
ICAM-1	intercellular adhesion molecule-1
IDI	integrated discrimination improvement
IL-6	interleukin-6
IQR	interquartile range
LC-MS/MS	liquid chromatography-tandem mass spectrometry
LDL	low-density lipoprotein
LOX-1	lectin-like oxidized low-density lipoprotein receptor-1
MCP-1	monocyte chemoattractant protein-1
NADPH	nicotinamide adenine dinucleotide phosphate
NF-κB	nuclear factor-kappa B
NO	nitric oxide
NRI	net reclassification improvement
OR	odds ratio
oxLDL	oxidized low-density lipoprotein
PCSK9	proprotein convertase subtilisin/kexin type 9
PKC	protein kinase C
ROS	reactive oxygen species
sLOX-1	soluble lectin-like oxidized low-density lipoprotein receptor-1
T2DM	type 2 diabetes mellitus
TC	total cholesterol
TG	triglycerides
TGF-β	transforming growth factor-beta
UACR	urinary albumin-to-creatinine ratio
VCAM-1	vascular cell adhesion molecule-1
VDR	vitamin D receptor
VEGF	vascular endothelial growth factor
