# Supplementary material for: Serum sLOX-1 and 25-Hydroxyvitamin D3 Across the Glycemic Spectrum: Associations with Metabolic and Microvascular Parameters in Prediabetes and Type 2 Diabetes
Source: J Clin Med. 2026 Oct 15;15(20):7989. doi: 10.3390/jcm15207989 (PMC13706463; doi:10.3390/jcm15207989)
Supplement: Supplementary file 1 [file jcm-15-07989-s001.zip › Table_S1.pdf]

Table S1. Unadjusted and Age- and BMI-Adjusted Between-Group Comparisons for Primary Biomarkers (n = 80).

Biomarker	Comparison	Control median (IQR)	Comparison group median (IQR)	KW p-value	ε^2	Pairwise p[a]	Ratio of medians[b]	95% CI	p-value
sLOX-1 (ng/mL)	*Prediabetic vs Control*	3.50 (2.25–5.85)	10.75 (3.57–16.58)	**0.0115**	0.090	**0.0397**	**2.91**	**1.05–8.07**	**0.040**
sLOX-1 (ng/mL)	*T2DM vs Control*	3.50 (2.25–5.85)	8.83 (3.21–23.26)	**0.0115**	0.090	**0.0209**	1.81	0.63–5.20	0.265
25(OH)D₃ (ng/mL)	*Prediabetic vs Control*	15.93 (10.64–19.15)	22.69 (15.69–30.97)	**0.0036**	0.117	0.4009	1.39	0.85–2.27	0.185
25(OH)D₃ (ng/mL)	*T2DM vs Control*	15.93 (10.64–19.15)	12.11 (7.59–19.74)	**0.0036**	0.117	0.2591	**0.59**	**0.35–0.97**	**0.039**
UACR (mg/g creatinine)	*Prediabetic vs Control*	4.99 (4.00–5.42)	7.47 (4.27–16.91)	**< 0.001**	0.328	**0.0331**	1.88	0.96–3.69	0.067
UACR (mg/g creatinine)	*T2DM vs Control*	4.99 (4.00–5.42)	18.15 (8.15–34.29)	**< 0.001**	0.328	**< 0.001**	**3.81**	**1.90–7.67**	**0.001**

[a] *Pairwise p-values from Dunn's post-hoc test (GraphPad Prism, v10); bold values indicate statistical significance ($p < 0.05$). For 25(OH)D_3, neither the prediabetic nor the T2DM group differed significantly from controls in the unadjusted analysis ($p = 0.4009$ and $p = 0.2591$, respectively); the significant comparison was T2DM vs prediabetic ($p = 0.0024$), which is not shown in this table as it focuses on group vs. control comparisons consistent with the quantile regression models.*

[b] *Ratio of medians (back-transformed from log scale) from quantile regression ($q = 0.50$) on log-transformed biomarker values, adjusting for age (years) and BMI (kg/m²) as continuous covariates. A ratio > 1 indicates higher values in the comparison group relative to controls; a ratio < 1 indicates lower values. 95% CI: 95% confidence interval. Bold values indicate FDR-significant correlations ($p < 0.05$). KW: Kruskal–Wallis; ε^2: epsilon-squared effect size (0.04 = small, 0.16 = medium, 0.36 = large); IQR: interquartile range; sLOX-1: soluble lectin-like oxidized low-density lipoprotein receptor-1; 25(OH)D_3: 25-hydroxyvitamin D_3; UACR: urinary albumin-to-creatinine ratio; T2DM: type 2 diabetes mellitus. Note: the limited age overlap between the control group (range 29–51 years) and the T2DM group (range 40–69 years), with only 38% of controls falling within the T2DM age range, constrains the reliability of these adjusted estimates. Adjusted analyses should therefore be interpreted as sensitivity analyses rather than definitive conclusions.*
