# Supplementary material for: Serum sLOX-1 and 25-Hydroxyvitamin D3 Across the Glycemic Spectrum: Associations with Metabolic and Microvascular Parameters in Prediabetes and Type 2 Diabetes
Source: J Clin Med. 2026 Oct 15;15(20):7989. doi: 10.3390/jcm15207989 (PMC13706463; doi:10.3390/jcm15207989)
Supplement: Supplementary file 1 [file jcm-15-07989-s001.zip › Table_S2.pdf]

Table S2. Unadjusted and Age-Adjusted Partial Spearman Rank Correlation Coefficients for Primary Biomarkers (n = 80).

Variable	Outcome	Unadjusted			Age-adjusted (partial r_p)		
		r	p	q (FDR)	r_p	p	q (FDR)
Section A – Biomarker correlations with metabolic parameters (corresponding to Table 2)							
sLOX-1	*Glucose*	+0.202	0.073	0.139	+0.032	0.820	0.820
sLOX-1	*Triglycerides*	+0.264	0.018	**0.047**	+0.182	0.106	0.171
sLOX-1	*Total Cholesterol*	-0.044	0.699	0.773	-0.162	0.226	0.226
sLOX-1	*HDL-cholesterol*	-0.173	0.124	0.174	-0.190	0.171	0.171
25(OH)D_3	*Glucose*	-0.220	0.050	0.106	-0.380	0.003	**0.003**
25(OH)D_3	*Triglycerides*	+0.019	0.868	0.868	-0.039	0.810	0.810
25(OH)D_3	*Total Cholesterol*	+0.187	0.097	0.165	+0.140	0.301	0.301
25(OH)D_3	*HDL-cholesterol*	+0.264	0.018	**0.047**	+0.265	0.018	0.058
UACR	*Glucose*	+0.485	< 0.001	**< 0.001**	+0.412	0.002	**0.002**
UACR	*Triglycerides*	+0.363	0.001	**0.006**	+0.303	0.006	**0.026**
UACR	*Total Cholesterol*	+0.257	0.021	**0.050**	+0.186	0.171	0.171
UACR	*HDL-cholesterol*	-0.084	0.457	0.565	-0.094	0.506	0.506
Section B – Biomarker correlations with waist circumference, HbA1c, and inter-biomarker pairs (corresponding to Table 3)							
sLOX-1	*Waist circumference*	+0.346	0.002	**0.007**	+0.221	0.049	0.111
sLOX-1	*HbA1c*	+0.296	0.008	**0.025**	+0.119	0.295	0.387
sLOX-1	*25(OH)D_3*	+0.054	0.633	0.738	-0.006	0.955	0.955
sLOX-1	*UACR*	+0.021	0.851	0.868	-0.080	0.479	0.559
25(OH)D_3	*Waist circumference*	-0.094	0.405	0.531	-0.217	0.052	0.111
25(OH)D_3	*HbA1c*	-0.180	0.110	0.165	-0.387	< 0.001	**0.003**
25(OH)D_3	*UACR*	-0.182	0.106	0.165	-0.248	0.027	0.070
UACR	*Waist circumference*	+0.359	0.001	**0.006**	+0.261	0.019	0.058
UACR	*HbA1c*	+0.555	< 0.001	**< 0.001**	+0.506	< 0.001	**< 0.001**

r: unadjusted Spearman rank correlation coefficient. r_p: age-adjusted partial Spearman rank correlation coefficient, computed by regressing ranks of each variable on age ranks and correlating the residuals. p: raw two-tailed p-value. q (FDR): Benjamini–Hochberg false discovery rate-adjusted p-value, computed separately across all tests in Section A and B combined. Bold q-values indicate FDR-significant correlations (q < 0.05). Age was treated as a continuous covariate. The partial correlation approach was applied to address the substantial age difference between the control group (median 38 years) and the dysglycemic groups (median 51–55 years). UACR: urinary albumin-to-creatinine ratio; sLOX-1: soluble lectin-like oxidized low-density lipoprotein receptor-1; 25(OH)D_3: 25-hydroxyvitamin D_3; HbA1c: glycated haemoglobin; HDL: high-density lipoprotein.
