# Supplementary material for: Serum sLOX-1 and 25-Hydroxyvitamin D3 Across the Glycemic Spectrum: Associations with Metabolic and Microvascular Parameters in Prediabetes and Type 2 Diabetes
Source: J Clin Med. 2026 Oct 15;15(20):7989. doi: 10.3390/jcm15207989 (PMC13706463; doi:10.3390/jcm15207989)
Supplement: Supplementary file 1 [file jcm-15-07989-s001.zip › Table_S3.pdf]

Table S3. Multicollinearity Assessment and Sensitivity Analyses for Logistic Regression Models (5-Predictor Specification).

Variable		Model 1: T2DM vs Control + Prediabetic (n = 80, events = 27, EPV = 5.4)		Model 2: Prediabetic vs Control (n = 53, events = 27, EPV = 5.4)	
	**VIF***	**OR (95% CI)**	**p-value**	**OR (95% CI)**	**p-value**
Covariates (pre-specified)					
Age (years)	1.321	1.111 (1.036–1.192)	**0.003**	1.159 (1.028–1.306)	**0.016**
Waist circumference (cm)	1.611	0.997 (0.954–1.041)	0.882	1.082 (0.993–1.178)	0.072
Primary predictors of interest					
25(OH)D_3 (ng/mL)	1.112	0.922 (0.861–0.987)	**0.020**	1.068 (0.972–1.174)	0.169
log(UACR)†	1.157	4.014 (1.793–8.986)	**0.001**	3.250 (0.917–11.516)	0.068
sLOX-1 (ng/mL)	1.216	1.058 (0.982–1.139)	0.136	1.018 (0.896–1.157)	0.778
**Model fit:** Model 1: AIC = 73.19, McFadden R^2 = 0.402, apparent AUC = 0.899 (95% CI: 0.843–0.899), optimism-corrected AUC = 0.871. Model 2:AIC = 42.66, McFadden R^2 = 0.583					

** VIF values < 5 indicate no concern; 5–10 indicate moderate but acceptable collinearity; > 10 indicate potentially problematic multicollinearity. Condition number < 30 indicates acceptable collinearity. The model predictors: age, waist circumference, 25(OH)D_3, log(UACR), and sLOX-1. BMI was excluded owing to high collinearity with waist circumference (Spearman r = 0.870); sex was excluded to preserve EPV. All VIF values are substantially lower in the model compared to the original 7-predictor model, reflecting improved predictor independence.*

†UACR was log-transformed to raw UACR (skewness = 7.39, maximum = 755.1 mg/g). The OR for log(UACR) represents the multiplicative change in odds per one-unit increase in log(UACR). Cook's distance analysis identified seven influential observations in Model 1 (Cook's D > 0.050); sensitivity analysis excluding these observations showed that the 25(OH)D_3 association remained significant (OR = 0.761, p = 0.011), whereas the log(UACR) estimate became extreme (OR > 700), indicating substantial sensitivity to observations with very high UACR values (> 100 mg/g). Bold p-values indicate statistical significance ($p < 0.05$). Predictor selection: BMI was excluded owing to high collinearity with waist circumference (Spearman r = 0.870); sex was excluded to reduce predictor count given low EPV. HbA1c was excluded due to complete separation between groups (T2DM range 6.5–12.9%; Control range 4.5–5.6%). EPV = 5.4 (27 events, 5 predictors) remains below the recommended minimum of 10; all estimates are therefore exploratory and hypothesis-generating. All 80 participants had complete data on all model variables; no imputation was required. Firth's penalized logistic regression was considered but could not be implemented in the available software and is recommended for future analyses. EPV: events per variable; AIC: Akaike Information Criterion; OR: odds ratio; CI: confidence interval; UACR: urinary albumin-to-creatinine ratio; sLOX-1: soluble lectin-like oxidized low-density lipoprotein receptor-1; 25(OH)D_3: 25-hydroxyvitamin D_3; T2DM: type 2 diabetes mellitus; BMI: body mass index.
